# Severe and prolonged febrile agranulocytosis under thyrosol

**DOI:** 10.1186/1471-2334-14-S7-P96

**Published:** 2014-10-15

**Authors:** Mihaela Ionică, Roxana Geantă, Șerban Benea, Bogdana Manu, Alina Cozma, Mihai Olariu, Olga Dorobăț, Cleo Roşculeț, Ramona Zamfir, Adrian Miron, Elisabeta Benea

**Affiliations:** 1 National Institute for Infectious Diseases "Prof. Dr. Matei Balş", Bucharest, Romania; 2 Elias University Emergency Hospital, Bucharest, Romania

## Background

It is well known that patients with severe neutropenia are susceptible to bacterial infections, which may become life-threatening. This hematologic disorder frequently occurs as an adverse effect of certain drug therapies. One of them, currently encountered in practice, is therapy with antithyroid drugs. An infective source is identified in average in 20-30% of febrile neutropenia episodes. Often the only infection proof is bacteremia, documented in 10-25% of patients.

## Case report

We report the case of a patient known with Basedow-Graves disease, who developed a febrile agranulocytosis under thyrosol, and in which *Pseudomonas aeruginosa* was isolated from blood culture. Although the antibiotic treatment proved efficient and the patient recovered the neutropenia due to granulocyte colony-stimulating factor, the initial evolution was unfavorable, due to the impossibility of continuing antithyroid treatment and due to a heart rhythm disorder that appeared subsequently, on the patient’s background of mitral and aortic regurgitation. During hospitalization, a transfer to the intensive care department was necessary.

After the remission of agranulocytosis, the patient underwent total thyroidectomy, because of an absolute contraindication of ever using thyrosol therapy. At 3 months of follow-up, the patient is on thyroid substitution, and is stable.

## Conclusion

Despite an initially poor prognosis, the eventual evolution was favorable, through interdisciplinary cooperation between infectious diseases, endocrinology, hematology, cardiology, intensive care and surgery.

## Consent

Written informed consent was obtained from the patient for publication of this Case report and any accompanying images. A copy of the written consent is available for review by the Editor of this journal.

